# Changes in Nutrition and Physical Activity up to 4 Years Following a Hypertensive Pregnancy: A Blood Pressure Postpartum Longitudinal Sub-Study

**DOI:** 10.3390/nu18203443

**Published:** 2026-10-20

**Authors:** Yining Chen, Megan Gow, Katie Harris, Lynne Roberts, Alison Canty, Wendy Pickup, Natasha Hirschhorn-Edwards, Amanda Henry

**Affiliations:** 1 The George Institute for Global Health, UNSW Medicine and Health, Sydney, NSW 2031, Australia; chenyn21@mails.tsinghua.edu.cn (Y.C.); mgow@georgeinstitute.org.au (M.G.); kharris@georgeinstitute.org.au (K.H.); 2 Tsinghua Medicine, Tsinghua University, Beijing 100084, China; 3 School of Clinical Medicine, UNSW Medicine and Health, Sydney, NSW 2033, Australia; lynne.roberts2@health.nsw.gov.au; 4 School of Population Health, UNSW Medicine and Health, Sydney, NSW 2033, Australia; n.h.edwards@unsw.edu.au; 5 Department of Women’s and Children’s Health, St George Hospital, Sydney, NSW 2217, Australia; 6 Women’s Health Initiative Translational Unit (WHITU), South Western Sydney Local Health District, Sydney, NSW 2170, Australia; alison.canty@health.nsw.gov.au (A.C.); wendy.pickup@health.nsw.gov.au (W.P.)

**Keywords:** hypertension, pregnancy-induced, healthy lifestyle, diet, exercise, cardiovascular diseases, postpartum period, longitudinal studies

## Abstract

Background/Objectives: Postpartum lifestyle interventions may mitigate increased cardiovascular disease (CVD) risks following hypertensive disorders of pregnancy (HDP). The study aimed to examine longitudinal changes in post-HDP lifestyle behaviors and women’s perceptions towards healthy living. Methods: This study was a longitudinal sub-study of the 3-armed, multicenter Blood Pressure Postpartum (BP^2^) randomized controlled trial in post-HDP women, comprising different postpartum care/lifestyle intervention strategies 6–12 months postpartum and post-intervention observational follow-up to 3–4 years postpartum. The NSW Health Population Survey captured lifestyle variables 6–12 months postpartum. At 3–4 years post-HDP, the Australian Eating Survey and the International Physical Activity Questionnaire were alternatively used for diet and physical activity, respectively. Linear mixed-effects models analyzed daily consumption of vegetables/fruit, moderate-to-vigorous physical activity (MVPA), and perception scores of healthy living for motivators, barriers, and satisfaction. Results: In 180 participants, reported mean (SD) vegetable/fruit daily intake increased (vegetable servings/day: 2.6 (1.3), 6 months vs. 2.7 (1.4), 12 months vs. 4.2 (2.0), 3–4 years, *p* < 0.001; fruit servings/day: 1.7 (1.0), 6 months vs. 1.7 (1.0), 12 months vs. 2.2 (1.3), 3–4 years, *p* < 0.001), while accompanied by other unfavorable dietary changes. Reported median [IQR] MVPA increased (minutes/week, 330 [280], 6 months vs. 313 [331], 12 months vs. 515 [690], 3–4 years, *p* < 0.001). Motivators to engage in healthy behaviors decreased (*p* < 0.001), perceived barriers increased (*p* < 0.001), and satisfaction with diet and body appearance worsened over time (*p* < 0.001). Conclusions: Overall, women reported higher intake of both health-promoting and less health-promoting foods and higher MVPA; however, different tools were used for lifestyle measurement. Worsening perceptions were observed using the same instrument.

## 1. Introduction

Hypertensive disorders of pregnancy (HDP), including chronic hypertension (CH), gestational hypertension (GH), pre-eclampsia (PE), and PE superimposed on CH (PE + CH) [1,2], are leading causes globally of maternal morbidity and mortality [2,3]. Complicating 5–10% of pregnancies worldwide [4,5], HDPs represent a significant burden on the health care system, not only during pregnancy but also long-term, with women’s lifelong cardiometabolic risks increased post-HDP. For instance, the 10-year risk of major cardiovascular disease (CVD) was estimated at 5.5 per 1000 in women following HDP, compared to 2.1 per 1000 in women without a HDP history [5]. Adopting lifestyle behaviors that support cardiovascular health is widely recommended in clinical practice to reduce HDP-associated CVD risks [6] by an estimated 10% [7].

Few randomized controlled trials (RCTs) have studied the effect of healthy lifestyle interventions on HDP-related CVD risks, with limited evidence supporting effective postpartum lifestyle management [8,9,10,11,12,13]. Short follow-up time is a common limitation of these trials. Previous observational studies have reported suboptimal lifestyle behaviors among women in the early postpartum period after HDP-affected pregnancies, including a greater prevalence of nutritional inadequacy [14] and lower physical activity levels [15] at six months postpartum compared to normotensive women.

Women after HDP also have significant barriers to maintaining long-term healthy lifestyle changes during the challenges of new parenthood [16,17]. Although not well understood, women’s motivators to engage in healthy behaviors and their level of satisfaction with their current dietary habits and body appearance may significantly influence their decisions to adopt healthier lifestyles [18,19].

While cross-sectional evidence indicates suboptimal diet quality after HDP and significant barriers to making lifestyle changes, longitudinal changes in lifestyle behaviors and perceptions towards healthy lifestyles following HDP remain unclear. Understanding how lifestyle behaviors and women’s motivators, barriers and perceptions regarding lifestyle change track over time is essential to inform better-structured postpartum lifestyle management in this post-HDP population.

The Blood Pressure Postpartum (BP^2^) study, a randomized controlled trial (RCT), evaluated the effect of three distinct lifestyle intervention strategies during the first 12 months post-HDP on reducing maternal CVD risks [20]. Previous cross-sectional BP^2^ sub-studies observed suboptimal lifestyle behaviors among post-HDP women [21,22]. Building on these findings, the aim of this study is to examine longitudinal changes in women’s self-reported lifestyle behaviors and perceptions of healthy living across the first 3–4 years postpartum. A secondary aim is to compare trajectories of lifestyle change across key subgroups: HDP subtype, baseline body mass index (BMI) category, and BP^2^ intervention group.

## 2. Materials and Methods

### 2.1. Study Design and Objectives

The BP^2^ trial, established in 2019, was a three-armed, multicenter RCT conducted across six metropolitan hospitals in Sydney, Australia. As per the previously published study protocol, the BP^2^ main study primarily aimed to examine changes in women’s cardiometabolic measurements, with a targeted enrolment of 480 HDP women who (1) were ≥18 years old, (2) gave birth at one of the study hospitals during the study period, and (3) met International Society for the Study of Hypertension in Pregnancy (ISSHP) criteria [1] for HDP. Non-English speakers were encouraged to be involved via the use of interpreter services and translated study materials.

At six months post-HDP, consented and eligible participants were randomized 1:1:1 into three BP^2^ intervention groups:

1. Optimized usual care with general lifestyle guidance and visits to their general practitioners at 6 months postpartum.
2. Brief education intervention via specialized clinic risk assessments and physician and dietary counseling at six months postpartum.
3. Extended lifestyle intervention, same as Group 2, plus 6-month phone-based NSW Health Get Healthy Service health coaching, administered by dietitians and/or exercise physiologists.

This BP^2^ sub-study focused on longitudinal changes in women’s lifestyle behavior and their perceptions towards healthy living, which are components of secondary outcomes in the parent BP^2^ study. These outcomes were evaluated by repeated self-reports at three timepoints: (1) baseline data collection at six months postpartum, (2) end-of-intervention assessments 12 months postpartum (acceptable range 11–16 months), and (3) observational follow-up at 3–4 years postpartum (acceptable range 2.8–4 years). These acceptable windows were predefined for standardization purposes. Although participants were originally randomized in the parent BP^2^ trial, the present longitudinal sub-study is primarily observational and describes lifestyle trajectories, without estimating causal effects of the BP^2^ interventions.

Women included in this longitudinal study were participants in the parent BP^2^ trial who completed lifestyle assessments at all three timepoints. The analysis cohort was selected to examine the trajectories of diet and physical activity within the same group of women. Women were excluded from this study if they (1) had no assessment of either diet or physical activity status, or both, at any timepoint, (2) were incorrectly randomized at six months post-HDP, (3) did not report baseline BMI data, or (4) completed assessments outside the acceptable timeframes.

### 2.2. Outcomes

The co-primary lifestyle outcomes, collected at three timepoints (6 months, 12 months, and 3–4 years postpartum), were:

1. Estimated daily intake (servings) of vegetables and fruit;
2. Estimated weekly duration (minutes/week) of moderate-to-vigorous intensity physical activity (MVPA).

For reference, a standard serve is defined as approximately 75 g (100–350 kJ) for vegetables and 150 g (350 kJ) for fruit [23]. Additionally, the proportion of women meeting the National Health and Medical Research Council dietary recommendation (≥5 vegetable servings/day and/or ≥2 fruit servings/day) [23] was assessed. Physical activity level was assessed based on adherence to the recommended ≥150 min of MVPA per week [24]. Other secondary lifestyle outcomes included daily intake of other food types (processed meat, fried potatoes, salty snacks, takeaway food, soft drinks and cordials, fruit drinks, and water), weekly duration of specific types of MVPA (walking, vigorous gardening work, and exercise during leisure time), smoking frequency, and alcohol consumption.

Women’s perceptions were analyzed across three domains: motivators to engage in healthy behaviors; barriers to adopting a healthy lifestyle; and satisfaction with food (taste and appetite) and with their body appearance, at each timepoint.

### 2.3. Data Collection

We used pre-existing self-administered questionnaires to evaluate women’s diet and physical activity at 6 months, 12 months, and 3–4 years post-HDP. Women’s perceptions towards healthy living were assessed using a study-specific survey at each timepoint. Further details are provided in the next sections.

#### 2.3.1. New South Wales (NSW) Population Health Survey (6- and 12-Months Nutrition and Physical Activity)

The NSW Population Health Survey is an annual survey collecting health behavior information of adults and children living in New South Wales [25]. In this study, we used the 2016 version of the survey conducted by telephone to collect data on nutrition, physical activity, smoking, and alcohol use.

#### 2.3.2. The Australian Eating Survey (3–4 Years Nutrition Data)

The online Australian Eating Survey (AES) [26] is a 120-item semi-quantitative Food Frequency Questionnaire (FFQ) estimating intake of each food type over the past 3–6 months using standardized portion sizes and/or natural serving sizes. To collect more comprehensive diet quality data than the NSW Population Health Survey, this study used the AES for women’s daily food intake at 3–4 years postpartum, with 19 questions directly related to vegetables and 11 to fruit. The estimated daily fruit intake was adjusted using the number of months per year when the fruit was available, with data obtained from Sydney produce markets and supermarket literature [27].

#### 2.3.3. International Physical Activity Questionnaire (3–4 Years Physical Activity)

To collect comprehensive physical activity using a questionnaire with results more readily comparable to international literature, a modified version of the International Physical Activity Questionnaire (IPAQ) long-form captured women’s frequency (days) and duration (minutes/day) of physical activities over the past 7 days before survey completion at 3–4 years postpartum. Weekly MVPA duration was estimated by aggregating ≥10 min continuous walking, moderate-intensity, and vigorous-intensity physical activities [28]. One question on moderate indoor chores was inadvertently omitted. Zero minutes spent on this physical activity was recorded for the cases with neither frequency nor duration for a specific question. Participants with valid frequency data but no duration were treated as missing cases.

#### 2.3.4. Healthy Lifestyle Statement Survey

A healthy lifestyle survey was designed to evaluate women’s perceptions of lifestyle behaviors on the basis of the ‘Adherence to a Healthy Lifestyle questionnaire’ [29]. This survey evaluates 18 statements across three domains: motivators to engage in healthy behaviors (5 statements), barriers to healthy living (9 statements), and satisfaction with food and appearance (4 statements). For each statement, women selected the response that best reflected their personal situation using a five-point Likert scale ranging from “not at all” to “extremely”. Responses were assigned ordinal scores from 1 to 5, respectively. Average scores of statements within each domain were categorized into a 1–5 scale and were used for descriptive and statistical analysis. Women were excluded from analyses for a specific domain if they provided an invalid response for any statement within that domain. In all domains, higher scores reflected greater levels of the concept being measured (although this survey was not validated): stronger motivation to engage in healthy behaviors, greater perceived barriers to healthy living, or higher satisfaction with food intake and body appearance.

### 2.4. Statistical Analysis

Descriptive analysis was initially performed to determine the distribution of the data using a combination of standard methods (Shapiro–Wilk test, skewness and kurtosis z-values, consideration of summary statistics, and visual inspection of frequency histograms and QQ-plots). Normally distributed continuous data are reported as mean ± standard deviation (SD), non-parametric continuous data as median [interquartile range (IQR, calculated as Q3-Q1)], and categorical or ordinal data as frequency (percentage).

Linear mixed-effects models (LMMs) were fitted for the longitudinal analysis of the co-primary and secondary continuous outcomes. To meet model assumptions, continuous variables that were not normally distributed were transformed to log10 (x + 1) form for statistical analyses and presentations. Generalized linear mixed models (GLMMs) were similarly applied to ordinal outcomes. *P*-values to assess the time effect from both LMM and GLMM were determined using a likelihood ratio test comparing the full model (with timepoint) to a reduced model (without timepoint). These LMMs or GLMMs were not adjusted for any covariates, as this analysis focuses on descriptive comparisons of the data. Pairwise comparisons for all models above were conducted between timepoints. For continuous outcomes, mean differences with standard errors (SE) were reported; for ordinal outcomes, odds ratios (OR) with 95% confidence intervals (CIs) were reported. All post hoc tests were adjusted for multiple comparisons using the Bonferroni method, with Bonferroni-adjusted *p*-values reported. No imputation was performed for missing values, and LMM/GLMM therefore used all available valid observations for each outcome.

To explore whether longitudinal trajectories differed between subgroups, interaction *p*-values representing time-by-subgroup effects were reported from models incorporating the following subgroup variables as exploratory analyses:

1. HDP subtype: CH, GH, PE, PE + CH as per ISSHP criteria [1];
2. Baseline BMI group: BMI ≥ 30 kg/m^2^ vs. <30 kg/m^2^ based on WHO obesity criteria;
3. BP^2^ intervention group: “Group 1” optimized usual care, “Group 2” brief education intervention, “Group 3” extended lifestyle intervention.

Sensitivity analyses were conducted for MVPA in women with complete physical activity measurements at each timepoint, regardless of completion of AES at 3–4 years post-HDP.

Statistical analyses were performed using R version 4.4.2 with the statistical significance level for this exploratory study set at *p* < 0.05.

### 2.5. Ethics Statement

The BP^2^ study received ethical approval from the South-Eastern Sydney Local Health District (Ref: 18/193, 25 October 2018). It was prospectively registered in the Australian and New Zealand Clinical Trials Registry (ACTRN12618002004246) on 13 December 2018, https://anzctr.org.au/Trial/Registration/TrialReview.aspx?id=376286&isReview=true (accessed on 17 October 2026). Informed consent was obtained from all participants in this study.

## 3. Results

**Figure 1:**
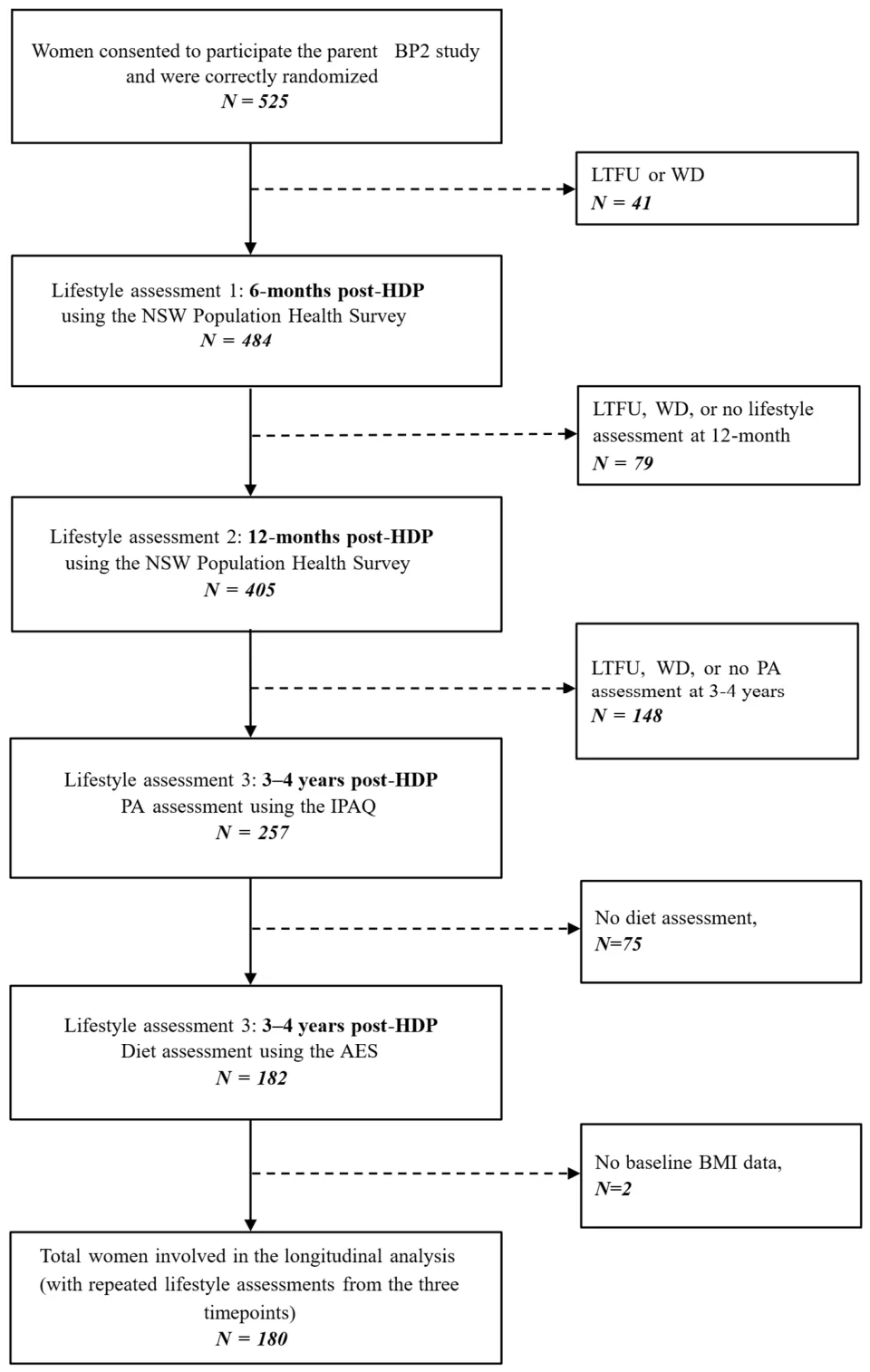
Flow chart of longitudinal lifestyle assessment from 6 to 12 months to 3–4 years post-HDP. LTFU, lost to follow-up; WD, withdrawn; PA, physical activity; AES, Australian Eating Surveys; IPAQ, International Physical Activity Questionnaire; BMI, body mass index; N, number.

Of the 525 women randomly assigned in BP^2^ at six months post-HDP, 484 women reported their diet and physical activity outcomes; of these, 405 participated at 12 months postpartum, and at the 3–4 years follow-up, 257 completed the IPAQ and 184 completed the AES. In total, 180 women had complete data at all three timepoints and were included in the longitudinal analysis (Figure 1).

### 3.1. Participant Characteristics and Baseline Status

Table 1 displays the characteristics of the 180 women included in this BP^2^ sub-study. Participants were 34.6 ± 4.7 years of age at the index birth, most were Australian-born (65%), identified as Caucasian ethnicity (65%), held a university degree (78%), were in a relationship at six months postpartum (95%), and had been diagnosed with preeclampsia (59%) during pregnancy. The parent study randomized 50 of these 180 women into BP^2^ intervention group 1 (28%), 66 into group 2 (37%), and 64 into group 3 (36%). Three lifestyle assessments occurred at a median of 0.5 [IQR 0.1], 1.0 [IQR 0.1], and 3.2 [IQR 0.4] years postpartum.

Compared to 345 excluded women, the 180 participants included in this study had a higher level of education (*p* < 0.001, Table S1), were less likely to have a history of diabetes (*p* = 0.003), were more likely to have had a planned index pregnancy (*p* = 0.01), were more likely to have sustained breastfeeding until at least six months postpartum (*p* = 0.001), and had a lower baseline BMI (*p* < 0.001). These 180 participants accounted for 34% of total women who were correctly randomized from the BP^2^ parent study, and 44% of women who completed the BP^2^ RCT at 12 months post-HDP.

### 3.2. Nutrition

From 6 months to 3–4 years postpartum, women reported significantly higher daily intake of vegetable servings (2.6 ± 1.3, 6 months vs. 2.7 ± 1.4, 12 months vs. 4.2 ± 2.0, 3–4 years, *p* < 0.001, Table 2, Figure S1) and fruit servings (1.7 ± 1.0, 6 months postpartum vs. 1.7 ± 1.0, 12 months vs. 2.2 ± 1.3, 3–4 years, *p* < 0.001). This represents a higher report of approximately 1.5 vegetable servings and 0.5 fruit servings at 3–4 years compared to earlier timepoints (all *p* < 0.001, Table S2). The proportion of women meeting the recommended daily vegetable intake (≥5 servings/day) tripled from approximately 10% during the first year to 36% at 3–4 years post-HDP (*p* < 0.001), while the proportion meeting fruit recommendations (≥2 servings/day) remained stable. Only 7% of participants (*n* = 12) met the dietary recommendations for both vegetables and fruit at 6–12 months, which increased to 26% at 3–4 years postpartum (*p* < 0.001).

At 3–4 years, intake of fried potato, salty snacks, soft drinks or cordials, and takeaway food frequencies were also higher compared to earlier timepoints, (*p* = 0.008 for soft drinks/cordials, *p* < 0.001 for others), while intake of water (*p* < 0.001) and fruit drinks (*p* = 0.006) were reduced.

From exploratory sub-group analyses, the BP^2^ intervention groups showed different longitudinal patterns for fruit and fried potato intake, and women with different HDP subtypes showed different patterns for soft drinks consumption, despite comparable cross-sectional results at each timepoint (Table 2 and Table S3–S5). No subgroup interaction effect was observed in the longitudinal changes in women’s diet patterns across baseline BMI.

### 3.3. Physical Activity Status

Median weekly MVPA did not change significantly during the BP^2^ intervention (330 [IQR 280] min/week at 6 months postpartum vs. 313 [IQR 331] min/week at 12 months) (Table 2). A significantly longer MVPA duration was reported at the 3–4 years follow-up compared with earlier timepoints (*p* < 0.001, Figure S1), with the median MVPA time reaching 515 [IQR 690] minutes per week (Table 2). Women with different HDP subtypes showed different longitudinal patterns for MVPA (*p* = 0.03), with no significant subgroup interaction effects observed across baseline BMI and BP^2^ intervention groups.

Walking time and leisure-time exercise did not change significantly over the study. However, women reported significantly more time on vigorous gardening work (*p* < 0.001), which more than doubled from the first year to 3–4 years post-HDP (Table S2). No subgroup differences were found for the longitudinal changes in specific MVPA types.

There were 75 women with complete physical activity data that were excluded in the primary analyses due to having an incomplete nutrition survey at 3–4 years postpartum. Compared to the 180 study participants in the primary analyses, those excluded women reported a greater improvement in total MVPA (Table S6, *p* < 0.001), similar to the observed increased MVPA trajectory in 180 included women, and a significantly increased walking time (*p* = 0.04) within the first 4 years post-HDP.

### 3.4. Other Lifestyle Outcomes and Women’s Perceptions

Although no significant longitudinal changes were observed for smoking status, we found significant changes in the frequency of alcohol consumption across the timepoints (*p* = 0.0498, Table 2). Pairwise comparisons found modestly increased alcohol consumption frequency at 12 months (OR = 1.60, 95%CI: 1.01–2.55) and 3–4 years (OR = 1.68, 95%CI: 1.06–2.66) compared to six months post-HDP. However, Bonferroni-adjusted *p*-values were not significant (Table S2). Overall time effect of alcohol consumption was weak after correction for multiple comparisons. No significant group interaction effects were observed for either smoking status or alcohol consumption.

**Figure 2:**
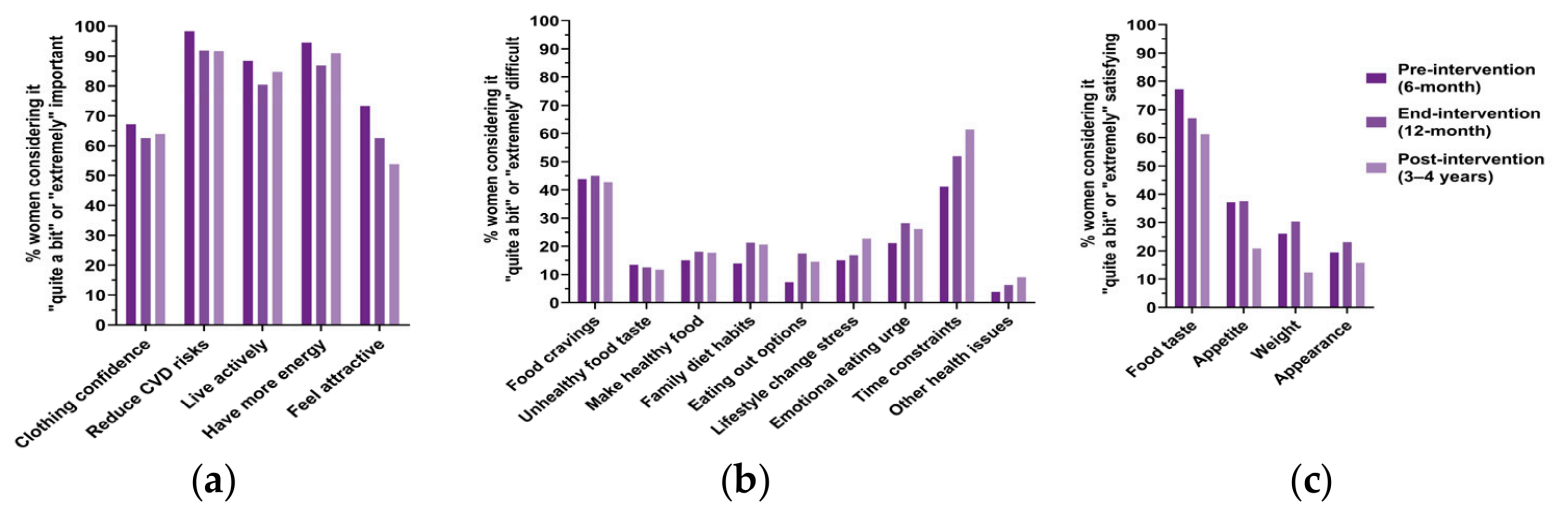
Longitudinal changes in women’s perceptions towards healthy living. Proportions of women considering lifestyle perception statements “quite a bit” or “extremely” applying to their personal situation regarding (**a**) motivators to engage in healthy behaviors; (**b**) barriers to healthy living; (**c**) satisfaction with their food and personal appearance are presented.

Women’s perceptions towards lifestyle interventions changed significantly, although modestly in absolute terms, over time (all *p* < 0.001, Table 2). Motivators to engage in healthy behaviors decreased, perceived barriers increased, and satisfaction with food and appearance declined. Despite this, overall motivation remained relatively high, with CVD risk reduction consistently identified as a primary motivator. Barriers to healthy living were generally perceived as low, though the greatest challenge shifted from food cravings to time constraints by 12 months postpartum. Satisfaction with food and body/personal appearance was consistently low, with the least satisfying aspect shifting from appearance to weight at 3–4 years postpartum (Figure 2).

## 4. Discussion

This BP^2^ sub-study provides longitudinal insights into nutrition, physical activity, smoking, alcohol consumption, and lifestyle perceptions among women during the period from 6 months to 3–4 years after HDP-affected pregnancies. Although some positive trends were observed in women’s daily intake of vegetables/fruit and MVPA, intake of vegetables/fruit remained below recommendations for most women. Motivation to engage in healthy behaviors reduced over time, while perceived barriers increased and satisfaction with appearance declined.

### 4.1. Long-Term Postpartum Lifestyle After HDP

Women reported a higher intake of vegetables and fruit and longer MVPA duration at 3–4 years postpartum compared with previous assessments. Time spent on vigorous gardening work more than doubled and accounted for the majority of the improvement seen in total time spent in MVPA, which was not affected by seasonal changes due to the rolling recruitment nature of this study. However, this finding may be a result of the change in assessment tool for MVPA, with the IPAQ long form known to inflate estimates [30,31].

Despite positive changes in primary outcomes, this was accompanied by unfavorable dietary changes, including decreased daily water intake and increased intake of less nutritious foods, including fried and salty snacks, sugary drinks, takeaway foods, and alcohol consumption. In contrast to higher reported intake of both health-promoting and less health-promoting foods, higher MVPA at 3–4 years should be interpreted cautiously, as it was largely driven by vigorous gardening activity, whereas walking and leisure-time exercise remained relatively unchanged. However, due to methodological biases, these findings for both diet and physical activity need to be interpreted with caution, since different tools were used to assess diet and physical activity at 3–4 years compared with 6 and 12 months postpartum; therefore, self-reported findings may not be directly comparable. Compared with the short questions from the NSW Population Health Survey, the more comprehensive AES, derived from FFQs, may lead to overestimation of daily food intake at 3–4 years post-HDP due to its longer recall period (past 3–6 months) and detailed nature.

Comparing our findings with prior evidence is challenging due to the limited number of long-term longitudinal studies in postpartum women, with most reported studies tracking lifestyle behaviors across the transition to motherhood [32,33] or within the first year postpartum [33,34]. One study of 473 Australian women observed stable diet quality from pregnancy to 3.5 years postpartum [35], while another found poorer dietary quality among 3434 women beyond 12 months postpartum compared to 1105 women < 12 months postpartum [36]. Our findings on improved fruit and vegetable intakes contrast with those of previous studies.

From 6 months to 3–4 years postpartum, women reported a decline in motivators to engage in healthy lifestyle behaviors and reductions in satisfaction with food and body appearance. Additionally, women reported increased barriers to maintaining a healthy lifestyle, with time constraints becoming the biggest challenge to overcome beyond 12 months postpartum. Although statistically significant, these changes were modest, and their clinical or behavioral relevance remains uncertain. These findings align with prior reports on postpartum women’s perceptions of healthy living motivations, barriers, and satisfaction [17,37,38].

The potential implications of these lifestyle changes for CVD risk remain speculative and require confirmation in studies incorporating clinical cardiovascular outcomes. Further research is required to determine structured and tailored postpartum lifestyle interventions that reduce CVD risks for women after HDP, but also give significant consideration to the motivators and barriers experienced by women at this life stage. Early structured support may need to be followed by booster or continuing support after 1 year postpartum to sustain healthy behaviors in this population.

### 4.2. Strengths and Limitations

The longitudinal analysis of this study, based on the parent BP^2^ study design, provides a comprehensive description beyond previous cross-sectional studies on women’s lifestyle changes in the first several years after HDP. Quantitative analysis of women’s perceptions towards a healthy lifestyle explored detailed motivation, barriers and drivers behind lifestyle behaviors, which can be utilized to improve women’s adherence in future post-HDP lifestyle intervention studies.

Some participants were 6–12 months post-HDP during periods of COVID-19 lockdown. Although this may have influenced lifestyle behaviors, our previous work found no significant effect of lockdowns on diet or physical activity among BP^2^ women following HDP, suggesting limited impact on the present findings [39]. Self-report surveys used in this study may introduce recall bias that potentially overestimates outcomes [40] and may have affected the observed behavioral changes over time. In short questionnaires such as the NSW Population Health Survey, previous research suggests that only one quarter of respondents report the same servings consumed for fruit and vegetables as reported in 24–hour recall or food diary measurement approach [41]. Use of AES at 3–4 years may overestimate dietary intake compared to 24–hour recalls and the gold standard of weighed food records [26], and the long-form IPAQ is known to overestimate MVPA compared to objective measurements [42] or 7–day physical activity recalls [43]. In addition, self-reported estimates may not be directly comparable across timepoints, due to different instruments used at 3–4 years compared with 6 and 12 months postpartum. Therefore, part of the observed changes may reflect methodological differences rather than true behavioral change. While AES and IPAQ have their own limitations, they were chosen at 3–4 years post-HDP to obtain comprehensive results that are more readily comparable with international studies.

Some subgroup categories were small, particularly the PE + CH subgroup, limiting statistical power and precision for interaction analyses. Given the multiple subgroup and interaction analyses, these findings were considered exploratory. In addition, some significant findings may reflect chance and should be interpreted cautiously, due to numerous secondary outcomes and subgroup analyses. Longitudinal findings regarding women’s perceptions towards healthy living should be interpreted with caution, given the use of a study-specific healthy lifestyle survey which has not been validated in this post-HDP population. Therefore, we suggest future longitudinal studies should employ consistent, more objective measures of lifestyle outcomes, such as food diaries for nutrients and wearable accelerometers for physical activity estimation, acknowledging that such measures have their own limitations, including increased cost and/or participant measurement burden.

Findings may not be generalizable as only 180 women (34% of the parent BP^2^ randomized participants) were included in this longitudinal analysis. In addition, included women had lower BMI, were better educated, and were more likely to breastfeed than excluded BP^2^ participants. These differences may have resulted in selection of a more health-engaged subgroup and may therefore have influenced the observed lifestyle trajectories. However, high attrition is common in postpartum studies due to time constraints, childcare priorities, and lack of support [44,45,46]. Therefore, although long follow-up is a key strength of our study, it should be balanced against substantial attrition across timepoints. Our study underscores the need for tailored, woman-centered postpartum lifestyle care models to improve women’s adherence.

The complete-case approach may have introduced selection bias, although it was used to examine dietary and physical activity trajectories within the same cohort. Sensitivity analysis showed a similarly increasing MVPA trajectory among the 75 excluded women; however, reporting and measurement bias may have contributed to this pattern. Therefore, representativeness of the presented findings may remain limited.

## 5. Conclusions

Using different assessment instruments at 3–4 years postpartum, women with prior HDP reported higher intake of both health-promoting and less health-promoting foods as well as higher MVPA than at earlier assessments. Additionally, worsening perceptions of motivators, barriers and satisfaction with dietary habits and body appearance suggest that overall lifestyle behaviors remain suboptimal. Consistent, validated measurement tools and targeted, long-term lifestyle interventions are needed to support behavior change and reduce CVD risk after HDP.

## Figures and Tables

**Table 1:** Maternal baseline data (recorded six months postpartum) of study participants.

Characteristics, Mean ± SD or Median [IQR] or *n* (%)	Participants (*n* = 180)
Maternal age at birth	34.6 ± 4.7
Born in Australia	117(65.0)
Ethnicity	
Caucasian	117 (65.0)
Asian	28 (15.6)
Aboriginal or Torres Strait Islander	1 (0.6)
Polynesian	0 (0.0)
European	13 (7.2)
Middle eastern	4 (2.2)
African	2 (1.1)
Mixed ^†^	11 (6.1)
Other	4 (2.2)
Education level	
Secondary school	8 (4.4)
Trade/certificate/diploma	31 (17.2)
University degree	141 (78.3)
In a relationship at 6 months postpartum	171 (95.0)
BP^2^ randomization at 6 months postpartum	
Group 1 (Optimized Usual Care)	50 (27.8)
Group 2 (Brief education)	66 (36.7)
Group 3 (Extended lifestyle coaching)	64 (35.6)
Randomization and follow-up periods in the BP^2^ study	
Timepoint 1: postpartum time	0.5 [0.1]
Maternal age	35.2 ± 4.7
Timepoint 2: postpartum time	1.0 [0.1]
Maternal age	35.7 ± 4.7
Timepoint 3: postpartum time	3.2 [0.4]
Maternal age at 3–4 years postpartum	38.0 ± 4.7
Past medical history	
Autoimmune disease	8 (4.4)
Cardiac disease	7 (3.9)
Endocrine disease	42 (23.3)
Diabetes (type 1, 2, and GDM)	6 (3.3)
Renal disease	19 (10.6)
Hypertension	44 (24.4)
Chronic Hypertension	24 (13.3)
Gestational Hypertension	11 (6.1)
Preeclampsia	15 (8.3)
Difficulty getting pregnant/fertility issues	42 (23.3)
Index HDP subtypes	
CH	24 (13.3)
GH	41 (22.8)
PE	106 (58.9)
PE + CH	9 (5.0)
Index pregnancy complications other than HDP	68 (37.8)
Index GDM	28 (15.6)
Index gestation weeks at birth	38.0 [3.0]
Index birth was the first baby	128 (71.1)
Index birth was a twin birth	3 (1.7)
Index pregnancy was planned	155 (86.1)
Discharge days after index birth	6.0 [3]
Admission to ICU/ACU/HDU after index birth	32 (17.8)
Breastfeeding after index birth	
Yes, and lasted over 6 months	126 (70.0)
Yes, but stopped before 6 months	47 (26.1)
No	7 (3.9)
Baseline BMI at 6 months postpartum	25.4 ± 5.4
BMI level	
<18.5 kg/m^2^	3 (1.7)
18.5–24.9 kg/m^2^	107 (59.4)
25.0–29.9 kg/m^2^	39 (21.7)
≥30 kg/m^2^	31 (17.2)
Blood pressure at 6 months postpartum	
Systolic blood pressure at baseline	115.2 ± 14.1
Diastolic blood pressure at baseline	71.1 ± 11.5

^†^ For women who selected more than one ethnicity. Abbreviations: SD, standard deviation; IQR, interquartile range; *n*, number; GDM, Gestational Diabetes Mellitus; HDP, Hypertensive Disorders of Pregnancy; CH, chronic hypertension; GH, gestational hypertension; PE, pre-eclampsia; PE + CH, pre-eclampsia superimposed on chronic hypertension; ICU, Intensive Care Unit; ACU, Acute Care Unit; HDU, High Dependency Unit; BMI, body mass index.

**Table 2:** Lifestyle outcomes at 6 months, 12 months and 3–4 years postpartum.

Lifestyle Outcomes	Descriptive Analysis			Time Effect, *p*-Value *	Time × Subgroup Interaction, *p*-Value *		
	6-Months	12-Months	3–4 Years		HDP	BMI	BP^2^
Nutrition, mean ± SD or median [IQR]							
Vegetables, serves/day	2.6 ± 1.3	2.7 ± 1.4	4.2 ± 2.0	<0.001	0.12	0.94	0.74
Fruit, serves/day	1.7 ± 1.0	1.7 ± 1.0	2.2 ± 1.3	<0.001	0.87	0.87	0.03
Processed meat, serves/day	0.14 [0.29]	0.14 [0.29]	0.14 [0.20]	0.06	0.72	0.63	0.12
Fried potatoes, serves/day	0.14 [0.11]	0.07 [0.11]	0.13 [0.13]	<0.001	0.10	0.19	0.05
Salty snacks, serves/day	0.07 [0.14]	0.03 [0.14]	0.41 [0.46]	<0.001	0.50	0.05	0.08
Takeaway food, times/week	0.46 [0.77]	0.46 [1.00]	1.50 [1.00]	<0.001	0.41	0.23	0.38
Soft drinks/Cordials, cups/day	0.00 [0.29]	0.00 [0.21]	0.08 [0.56]	0.008	0.008	0.36	0.48
Fruit drinks, cups/day	0.00 [0.14]	0.00 [0.07]	0.02 [0.08]	0.006	0.82	0.58	0.72
Water, cups/day	7.00 [5.00]	6.00 [4.00]	5.00 [5.00]	<0.001	0.55	0.66	0.30
Physical activity, minutes/week, median [IQR]							
MVPA ^a^	330 [280]	313 [331]	515 [690]	<0.001	0.03	0.84	0.80
Walking ^b^	210 [176]	180 [226]	198 [268]	0.62	0.39	0.47	0.81
Vigorous gardening work ^c^	0 [0]	0 [0]	0 [20]	<0.001	0.11	0.55	0.87
Leisure time exercise ^c^	30 [135]	40 [150]	0 [120]	0.12	0.68	0.59	0.99
Scores of women’s perceptions towards lifestyle interventions, mean ± SD							
Motivators to engage in healthy behaviors ^d^	4.3 ± 0.6	4.1 ± 0.6	4.1 ± 0.6	<0.001	0.39	0.61	0.94
Barriers to healthy living ^e^	2.3 ± 0.6	2.5 ± 0.7	2.5 ± 0.7	<0.001	0.35	0.25	0.93
Satisfaction with food and appearance ^f^	3.0 ± 0.9	3.0 ± 0.8	2.7 ± 0.8	<0.001	0.73	0.13	0.71
Meet healthy lifestyle recommendations, *n* (%)							
≥5 vegetable serves/day	17 (9.4)	18 (10.0)	64 (35.6)	<0.001	0.81	0.49	0.68
≥2 fruit serves/day	93 (51.7)	97 (53.9)	93 (51.7)	0.85	0.91	0.65	0.24
≥5 vegetable and 2 fruit serves/day	13 (7.2)	13 (7.2)	47 (26.1)	<0.001	0.38	0.76	0.17
≥150 MVPA minutes/week ^a^	151 (91.5)	151 (91.5)	143 (86.7)	0.77	0.42	0.93	0.97
Meet both for nutrition and MVPA ^a^	12 (7.3)	13 (7.9)	42 (25.5)	<0.001	0.04	0.009	0.03
Smoking or using electronic cigarettes ^g^							
Current/previous	32 (17.8)	39 (21.8)	36 (20.1)	0.25	0.17	0.17	0.12
Tried a few times	38 (21.1)	35 (19.6)	36 (20.1)				
Never	110 (61.1)	105 (58.7)	107 (59.8)				
Alcohol drinking frequency ^g^							
4–7 days/week	7 (3.9)	7 (3.9)	13 (7.3)	0.05	0.58	0.49	0.67
1–3 days/week	50 (27.8)	61 (33.9)	43 (24.0)				
Less than 1 day/week	30 (16.7)	33 (18.3)	33 (18.4)				
Less than 1 day/month	26 (14.4)	18 (10.0)	42 (23.5)				
Never	67 (37.2)	61 (33.9)	48 (26.8)				

^a^ 15 missing; ^b^ 6 missing; ^c^ 4 missing; ^d^ 46 missing; ^e^ 25 missing; ^f^ 3 missing; ^g^ 1 missing. * *p*-values of timepoint effect, significant at *p* < 0.05, are determined using likelihood tests of LMMs or GLMMs. Interaction *p*-values, significant at *p* < 0.05, represent time-by-subgroup effects (whether longitudinal trajectories differed between subgroups) rather than simple between-group differences. Abbreviations: HDP, Hypertensive Disorders of Pregnancy; BMI, body mass index; SD, standard deviation; IQR, interquartile range; *n*, number.

## Data Availability

The datasets used and/or analyzed during the current study will be available from the corresponding author upon reasonable request after the main paper is published (3–4 years).

## Supplementary Materials

The following supporting information can be downloaded at https://www.mdpi.com/article/10.3390/nu18203443/s1, Figure S1: Longitudinal changes of primary lifestyle outcomes. Note: A) daily serves of vegetables (*p* < 0.001); B) daily serves of fruit (*p* < 0.001); C) MVPA weekly duration in log10(x + 1) form (*p* < 0.001, 15 missing). Black dots and boxes indicate actual responses, blue area represents the distribution pattern of outcomes, and red line represents the LMM predicted trajectories throughout the study process; Table S1: Comparison of maternal baseline data between participants included and excluded from BP^2^; Table S2: Pairwise comparison lifestyle outcomes at 6-months, 12-months and 3–4 years postpartum; Table S3: Subgroup analysis of women’s lifestyle outcomes at six months post-HDP stratified by hypertensive pregnancy subtype, body mass index, and study group; Table S4: Subgroup analysis of women’s lifestyle outcomes at 12 months post-HDP stratified by hypertensive pregnancy subtype, body mass index, and study group; Table S5: Subgroup analysis of women’s lifestyle outcomes at 3–4 years post-HDP stratified by hypertensive pregnancy subtype, body mass index, and study group; Table S6: Sensitivity analysis of physical activity status between study participants included and excluded at 3–4 years postpartum due to no diet reports from the Australia Eating Survey.

## Abbreviations

The following abbreviations are used in this manuscript:

AES	The Australian Eating Survey
BMI	Body Mass Index
BP^2^	The Blood Pressure Postpartum Study
CH	Chronic Hypertension
CVD	Cardiovascular disease
FFQ	Food Frequency Questionnaire
GH	Gestational Hypertension
HDP	Hypertensive Disorders of Pregnancy
IPAQ	The International Physical Activity Questionnaire
IQR	Interquartile Range
ISSHP	International Society for the Study of Hypertension in Pregnancy
MVPA	Moderate-to-Vigorous intensity Physical Activity
PE	Pre-eclampsia
PE + CH	Pre-eclampsia superimposed on chronic hypertension
RCT	Randomized Controlled Trials
SD	Standard Deviation
