# Supplementary material for: Changes in Nutrition and Physical Activity up to 4 Years Following a Hypertensive Pregnancy: A Blood Pressure Postpartum Longitudinal Sub-Study
Source: Nutrients. 2026 Oct 20;18(20):3443. doi: 10.3390/nu18203443 (PMC13708477; doi:10.3390/nu18203443)
Supplement: Supplementary file 1 [file nutrients-18-03443-s001.zip › nutrients-4554482-supplementary.pdf]

# Supplementary Materials

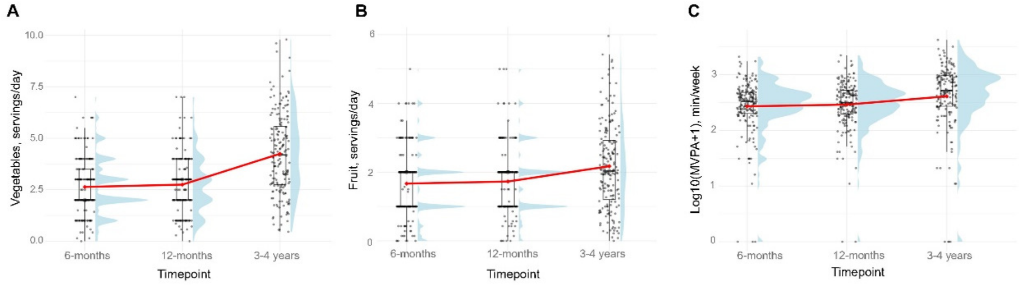


**Figure S1.** Longitudinal changes of primary lifestyle outcomes. Note: A) daily serves of vegetables (p<0.001); B) daily serves of fruit (p<0.001); C) MVPA weekly duration in log10(x+1) form (p<0.001, 15 missing). Black dots and boxes indicate actual responses, blue area represents the distribution pattern of outcomes, and red line represents the LMM predicted trajectories throughout the study process.

**Table S1**. Comparison of maternal baseline data between participants included and excluded from BP[2]

| Characteristics | Included (n=180) | Excluded (n=345) | *p*-Value |
|---|---|---|---|
| ***Demographical status, n (%)*** | | | |
| Maternal age when giving birth, mean±SD | 34.6±4.7 | 34.0±5.3 | 0.18 |
| Born in Australia | 117(65.0) | 196(56.8) | 0.09 |
| Ethnicity | | | |
| Caucasian | 117(65.0) | 190(55.6) | 0.05 |
| Asian | 28(15.6) | 58(17.0) | 0.77 |
| Aboriginal or Torres Strait Islander | 1(0.6) | 3(0.9) | 1.00 |
| Polynesian | 0(0.0) | 10(2.9) | 0.02* |
| European | 13(7.2) | 22(6.4) | 0.87 |
| Middle eastern | 4(2.2) | 8(2.3) | 1.00 |
| African | 2(1.1) | 10(2.9) | 0.23 |
| Mixed† | 11(6.1) | 17(5.0) | 0.73 |
| Other | 4(2.2) | 24(7.0) | 0.04* |
| Education level | | | |
| Secondary school | 8(4.4) | 43(12.6) | |
| Trade/certificate/diploma | 31(17.2) | 93(27.3) | <0.001*** |
| University degree | 141(78.3) | 205(60.1) | |
| In a relationship at 6-month postpartum | 171(95.0) | 309(90.6) | 0.11 |
| ***Randomisation and follow-up periods in the BP[2] study*** | | | |
| BP[2] randomisation at 6-month postpartum | | | |
| Group 1 (Optimised Usual Care) | 50(27.8) | 120(34.8) | |
| Group 2 (Brief education) | 66(36.7) | 115(33.3) | 0.27 |
| Group 3 (Extended lifestyle coaching) | 64(35.6) | 110(31.9) | |
| Timepoint 1: postpartum time, median [IQR] | 0.5[0.1] | 0.6[0.1] | N/A |
| Maternal age | 35.2±4.7 | 34.8±5.4 | N/A |
| Timepoint 2: postpartum time, median [IQR] | 1.0[0.1] | 1.1[0.1] | N/A |
| Maternal age | 35.7±4.7 | 35.2±5.4 | N/A |
| Timepoint 3: postpartum time, median [IQR] | 3.2[0.4] | 3.3[0.4] | N/A |
| Maternal age at 3-4 years postpartum | 38.0±4.7 | 38.1±5.7 | N/A |
| ***Past medical history, n (%)*** | | | |
| Autoimmune disease | 8(4.4) | 17(4.9) | 0.98 |
| Cardiac disease | 7(3.9) | 21(6.1) | 0.39 |
| Endocrine disease | 42(23.3) | 111(32.2) | 0.04* |
| Diabetes (type 1, 2, and GDM) | 6(3.3) | 39(11.3) | 0.003** |
| Renal disease | 19(10.6) | 37(10.7) | 1.00 |
| Hypertension | 44(24.4) | 106(30.7) | 0.16 |
| Chronic Hypertension | 24(13.3) | 61(17.7) | 0.25 |
| Gestational Hypertension | 11(6.1) | 21(6.1) | 1.00 |
| Preeclampsia | 15(8.3) | 40(11.6) | 0.31 |
| Difficulty conceiving a pregnancy or fertility issues | 42(23.3) | 78(22.7) | 0.97 |

| Characteristics | Included (n=180) | Excluded (n=345) | *p*-Value |
|---|---|---|---|
| ***Index pregnancy complications, n (%)*** | | | |
| HDP subtypes | | | |
| CH | 24(13.3) | 57(16.5) | 0.55 |
| GH | 41(22.8) | 89(25.8) | |
| PE | 106(58.9) | 181(52.5) | |
| PE+CH | 9(5.0) | 18(5.2) | |
| Had complications other than HDP | 68(37.8) | 134(38.8) | 0.89 |
| Index GDM | 28(15.6) | 62(18.0) | 0.57 |
| ***Index birth*** | | | |
| Gestation weeks at birth, median [IQR] | 38.0[3.0] | 38.0[3.0] | 0.58 |
| First baby | 128(71.1) | 223(64.6) | 0.16 |
| Twin birth | 3(1.7) | 13(3.8) | 0.29 |
| Planned pregnancy | 155(86.1) | 260(76.2) | 0.01* |
| Discharge days after birth, median [IQR] | 6.0[3] | 6.0[3.0] | 0.93 |
| Admission to ICU/ACU/HDU after birth | 32(17.8) | 51(14.8) | 0.44 |
| Breastfeeding | | | |
| Yes, and lasted over 6 months | 126(70.0) | 195(57.0) | 0.001** |
| Yes, but stopped before 6-month- | 47(26.1) | 104(30.4) | |
| No | 7(3.9) | 43(12.6) | |
| ***Baseline physical measurements, mean ± SD*** | | | |
| BMI | 25.4±5.4 | 27.8±6.9 | <0.001*** |
| BMI level, n (%) | | | |
| <18.5 kg/m$^2$ | 3(1.7) | 10(2.9) | <0.001*** |
| 18.5-24.9 kg/m$^2$ | 107(59.4) | 127(37.5) | |
| 25.0-29.9 kg/m$^2$ | 39(21.7) | 103(30.4) | |
| ⩾30 kg/m$^2$ | 31(17.2) | 99(29.2) | |
| Systolic BP | 115.2±14.1 | 116.3±15.0 | 0.43 |
| Diastolic BP | 71.1±11.5 | 71.6±9.7 | 0.62 |

†For women who selected more than one ethnicity.

*Significant at p<0.05, **significant at p<0.01, ***significant at p<0.001, using student's t-test for normally distributed variables, Wilcoxon rank-sum test for nonparametric variables, and Chi-square or Fisher's exact test for categorical variables.

*Abbreviations*: SD, standard deviation; IQR, interquartile range; n, number; GDM, Gestational Diabetes Mellitus; HDP, Hypertensive Disorders of Pregnancy; CH, chronic hypertension; GH, gestational hypertension; PE, pre-eclampsia; CH+PE, pre-eclampsia superimposed on chronic hypertension; ICU, Intensive Care Unit; ACU, Acute Care Unit; HDU, High Dependency Unit; BMI, body mass index; BP, blood pressure.

**Table S2**. Pairwise comparison lifestyle outcomes at 6months, 12months and 3-4 years postpartum.

| **Lifestyle outcomes** | **12-months vs. 6-months** | | | **3-4 years vs. 6-months** | | | **3-4 years vs. 12-months** | | |
|---|---|---|---|---|---|---|---|---|---|
| | Estimate[†] | SE | *p*-Value | Estimate[†] | SE | *p*-Value | Estimate[†] | SE | *p*-Value |
| **Nutrition** | | | | | | | | | |
| Vegetable, serves/day | 0.11 | 0.13 | 1.00 | 1.61 | 0.13 | <0.001[***] | 1.50 | 0.13 | <0.001[***] |
| ⩾5 vegetable serves/day | 1.09 | 0.45 | 1.00 | 11.06 | 4.62 | <0.001[***] | 10.17 | 4.16 | <0.001[***] |
| Fruit, serves/day | 0.06 | 0.09 | 1.00 | 0.51 | 0.09 | <0.001[***] | 0.45 | 0.09 | <0.001[***] |
| ⩾2 fruit serves/day | 1.13 | 0.27 | 1.00 | 1.00 | 0.24 | 1.00 | 0.89 | 0.22 | 1.00 |
| Meet both for vegetable and fruit | 1.00 | 0.48 | 1.00 | 9.82 | 5.12 | <0.001[***] | 9.82 | 5.12 | <0.001[***] |
| Processed meat, serves/day | -0.017 | 0.007 | 0.05 | -0.009 | 0.007 | 0.62 | 0.008 | 0.007 | 0.80 |
| Fried potatoes, serves/day | -0.013 | 0.005 | 0.03[*] | 0.009 | 0.005 | 0.20 | 0.023 | 0.005 | <0.001[***] |
| Salty snacks, serves/day | -0.014 | 0.007 | 0.14 | 0.096 | 0.007 | <0.001[***] | 0.110 | 0.007 | <0.001[***] |
| Takeaway food, times/week | -0.030 | 0.013 | 0.07 | 0.113 | 0.013 | <0.001[***] | 0.144 | 0.013 | <0.001[***] |
| Soft drinks/Cordials, cups/day | -0.018 | 0.009 | 0.12 | 0.009 | 0.009 | 0.93 | 0.027 | 0.009 | 0.007[**] |
| Fruit drinks, cups/day | -0.022 | 0.007 | 0.008[**] | -0.018 | 0.007 | 0.04[*] | 0.004 | 0.007 | 1.00 |
| Water, cups/day | -0.013 | 0.013 | 0.94 | -0.134 | 0.013 | <0.001[***] | -0.122 | 0.013 | <0.001[***] |
| **Physical activity** | | | | | | | | | |
| MVPA [a] | 0.032 | 0.044 | 1.00 | 0.180 | 0.045 | <0.001[***] | 0.149 | 0.045 | 0.003[**] |
| ⩾150 MVPA minutes/week | 1.06 | 0.36 | 1.00 | 1.28 | 0.45 | 1.00 | 1.20 | 0.42 | 1.00 |
| Meet both for nutrition and MVPA | 1.00 | 0.61 | 1.00 | 84.41 | 80.20 | <0.001[***] | 84.41 | 80.21 | <0.001[***] |
| Walking [b] | 0.036 | 0.052 | 1.00 | 0.050 | 0.053 | 1.00 | 0.014 | 0.053 | 1.00 |
| Vigorous gardening work | -0.029 | 0.066 | 1.00 | 0.327 | 0.067 | <0.001[***] | 0.356 | 0.067 | <0.001[***] |
| Leisure time exercise | 0.082 | 0.087 | 1.00 | -0.099 | 0.088 | 0.77 | -0.182 | 0.088 | 0.12 |

| **Lifestyle outcomes** | **12-months vs. 6-months** | | | **3-4 years vs. 6-months** | | | **3-4 years vs. 12-months** | | |
|---|---|---|---|---|---|---|---|---|---|
| | Estimate[†] | SE | *p*-Value | Estimate[†] | SE | *p*-Value | Estimate[†] | SE | *p*-Value |
| **Healthy lifestyle perceptions** | | | | | | | | | |
| Motivators to engage in healthy behaviours | -0.20 | 0.05 | <0.001[***] | -0.18 | 0.05 | 0.001[**] | 0.02 | 0.05 | 1.00 |
| Barriers to healthy living [†] | 0.18 | 0.05 | <0.001[***] | 0.17 | 0.05 | 0.001[**] | -0.02 | 0.05 | 1.00 |
| Satisfaction with food & appearance [†] | -0.04 | 0.06 | 1.00 | -0.31 | 0.06 | <0.001[***] | -0.27 | 0.06 | <0.001[***] |
| | OR | 95%CI | *p*-Value | OR | 95%CI | *p*-Value | OR | 95%CI | *p*-Value |
| **Other risky lifestyle behaviours** | | | | | | | | | |
| Smoking or using electronic cigarettes | 1.62 | [0.86, 3.05] | 0.41 | 1.34 | [0.71, 2.54] | 1.00 | 0.83 | [0.44, 1.55] | 1.00 |
| Alcohol drinking frequency [c] | 1.60 | [1.01, 2.55] | 0.14 | 1.68 | [1.06, 2.66] | 0.08 | 1.05 | [0.67, 1.65] | 1.00 |

[a]15 missing; [b]6 missing; [c]1 missing. [†]Estimates represent actual change of normally distributed data (vegetable, fruit and average domain scores of healthy lifestyle perceptions), or reveal the ratio change of other continuous outcomes using log10 (x+1) transformed value in LMM models.

[*]Significant at p<0.05, [**]significant at p<0.01, [***]significant at p<0.001.

*Abbreviations*: HDP, Hypertensive Disorders of Pregnancy; BMI, body mass index; SD, standard deviation; IQR, interquartile range; n, number.

**Table S3**. Subgroup analysis of women's lifestyle outcomes at six months post-HDP stratified by hypertensive pregnancy subtype, body mass index, and study group

| Lifestyle outcomes | HDP subtypes | | | | | Baseline BMI | | | BP[2] groups | | | |
|---|---|---|---|---|---|---|---|---|---|---|---|---|
| | CH (n=24) | GH (n=41) | PE (n=106) | PE+CH (n=9) | *p*-Value | <30 kg/m$^2$ (n=149) | ≥30 kg/m$^2$ (n=31) | *p*-Value | Group 1 (n=50) | Group 2 (n=66) | Group 3 (n=64) | *p*-Value |
| ***Mean ± SD*** | | | | | | | | | | | | |
| Vegetable, serves/day | 2.3±1.2 | 2.8±1.5 | 2.6±1.2 | 3.0±2.2 | 0.45 | 2.7±1.4 | 2.5±1.1 | 0.38 | 2.7±1.4 | 2.6±1.2 | 2.6±1.4 | 0.98 |
| Fruit, serves/day | 1.4±1.1 | 1.5±0.8 | 1.8±1.0 | 1.6±1.2 | 0.18 | 1.8±1.0 | 1.1±0.9 | <0.001*** | 1.8±1.0 | 1.8±1.0 | 1.5±1.0 | 0.16 |
| Motivators to engage in healthy behaviours | 4.4±0.5 | 4.3±0.5 | 4.2±0.6 | 4.3±0.6 | 0.82 | 4.3±0.6 | 4.3±0.6 | 0.90 | 4.3±0.5 | 4.2±0.6 | 4.3±0.6 | 0.58 |
| Barriers to healthy living [a] | 2.5±0.7 | 2.4±0.5 | 2.3±0.6 | 2.5±0.8 | 0.22 | 2.3±0.6 | 2.6±0.7 | 0.01* | 2.4±0.7 | 2.4±0.6 | 2.3±0.6 | 0.64 |
| Satisfaction with food & appearance | 2.6±0.7 | 3.1±0.7 | 3.1±0.9 | 3.1±1.0 | 0.02* | 3.2±0.8 | 2.3±0.6 | <0.001*** | 3.1±0.8 | 3.1±0.9 | 2.9±0.8 | 0.49 |
| ***Median [IQR]*** | | | | | | | | | | | | |
| **Diet** | | | | | | | | | | | | |
| Processed meat, serves/day | 0.14[0.31] | 0.14[0.26] | 0.14[0.29] | 0.00[0.14] | 0.32 | 0.14[0.29] | 0.14[0.40] | 0.12 | 0.14[0.25] | 0.14[0.36] | 0.12[0.29] | 0.32 |
| Fried potatoes, serves/day | 0.14[0.29] | 0.07[0.11] | 0.14[0.11] | 0.07[0.26] | 0.83 | 0.10[0.11] | 0.14[0.24] | 0.23 | 0.10[0.13] | 0.14[0.11] | 0.14[0.19] | 0.70 |
| Salty snacks, serves/day | 0.14[0.27] | 0.03[0.29] | 0.07[0.14] | 0.14[0.07] | 0.39 | 0.07[0.14] | 0.07[0.21] | 0.79 | 0.07[0.14] | 0.07[0.14] | 0.07[0.14] | 0.57 |
| Takeaway food, times/week | 1.00[0.54] | 0.69[0.77] | 0.46[0.77] | 0.00[0.34] | 0.01* | 0.46[0.77] | 1.00[0.89] | 0.86 | 1.00[0.77] | 0.46[0.77] | 0.57[0.77] | 0.41 |
| Soft drinks/Cordials, cups/day | 0.21[1.50] | 0.00[0.14] | 0.00[0.21] | 0.00[0.29] | 0.05 | 0.00[0.14] | 0.14[1.00] | 0.006** | 0.00[0.14] | 0.00[0.14] | 0.07[0.64] | 0.05 |
| Fruit drinks, cups/day | 0.00[0.21] | 0.00[0.00] | 0.00[0.20] | 0.00[0.00] | 0.55 | 0.00[0.14] | 0.00[0.07] | 0.55 | 0.00[0.14] | 0.00[0.29] | 0.00[0.14] | 0.88 |
| Water, cups/day | 7.00[6.50] | 7.00[3.00] | 7.00[3.88] | 8.00[3.00] | 0.43 | 7.00[3.00] | 6.00[5.50] | 0.54 | 7.00[4.00] | 7.50[5.00] | 7.00[3.50] | 0.95 |
| **Physical activity** | | | | | | | | | | | | |
| MVPA | 368[395] | 300[300] | 325 [251] | 390[250] | 0.93 | 330[290] | 360[265] | 0.65 | 300[296] | 310[255] | 390[296] | 0.51 |
| Walking | 210[200] | 200[210] | 195[160] | 240[120] | 0.74 | 210[180] | 200[165] | 0.43 | 210[198] | 185[190] | 210[165] | 0.86 |
| Vigorous gardening work | 0[0] | 0[0] | 0[0] | 0[0] | 0.22 | 0[0] | 0[0] | 0.45 | 0[0] | 0[0] | 0[0] | 0.62 |
| Leisure time exercise | 15[240] | 30[210] | 38[120] | 0[10] | 0.42 | 30[150] | 0[70] | 0.43 | 60[203] | 30[88] | 30[123] | 0.37 |

| Lifestyle outcomes | HDP subtypes | | | | | Baseline BMI | | | BP² groups | | | |
|---|---|---|---|---|---|---|---|---|---|---|---|---|
| | CH (n=24) | GH (n=41) | PE (n=106) | PE+CH (n=9) | *p*-Value | <30 kg/m² (n=149) | ≥30 kg/m² (n=31) | *p*-Value | Group 1 (n=50) | Group 2 (n=66) | Group 3 (n=64) | *p*-Value |
| ***Frequency (%)*** | | | | | | | | | | | | |
| ≥5 vegetable serves/day | 1(4.2) | 5(12.2) | 9(8.5) | 2(22.2) | 0.34 | 16(10.7) | 1(3.2) | 0.31 | 6(12.0) | 5(7.6) | 6(9.4) | 0.72 |
| ≥2 fruit serves/day | 8(33.3) | 19(46.3) | 63(59.4) | 3(33.3) | 0.06 | 86(57.7) | 7(22.6) | <0.001*** | 29(58.0) | 37(56.1) | 27(42.2) | 0.16 |
| Meet both for vegetable and fruit | 0(0.0) | 4(9.8) | 7(6.6) | 2(22.2) | 0.13 | 12(8.1) | 1(3.2) | 0.70 | 6(12.0) | 3(4.5) | 4(6.2) | 0.31 |
| ≥150 MVPA minutes/week | 19(79.2) | 34(82.9) | 91(85.8) | 7(77.8) | 0.70 | 127(85.2) | 24(77.4) | 0.42 | 41(82.0) | 55(83.3) | 55(85.9) | 0.84 |
| Meet both for nutrition and MVPA | 0(0.0) | 3(7.3) | 7(6.6) | 2(22.2) | 0.16 | 12(8.1) | 0(0.0) | 0.23 | 6(12.0) | 3(4.5) | 3(4.7) | 0.22 |
| Smoking or using e-cigarettes | | | | | | | | | | | | |
| Never | 11(45.8) | 21(51.2) | 70(66.0) | 8(88.9) | | 95(63.8) | 15(48.4) | | 32(64.0) | 43(65.2) | 35(54.7) | |
| Tried a few times | 6(25.0) | 9(22.0) | 22(20.8) | 1(11.1) | 0.12 | 32(21.5) | 6(19.4) | 0.07 | 7(14.0) | 18(27.3) | 13(20.3) | 0.05 |
| Current/previous | 7(29.2) | 11(26.8) | 14(13.2) | 0(0.0) | | 22(14.8) | 10(32.3) | | 11(22.0) | 5(7.6) | 16(25.0) | |
| Alcohol drinking frequency [c] | | | | | | | | | | | | |
| Never | 11(45.8) | 13(31.7) | 37(34.9) | 6(66.7) | | 56(37.6) | 11(35.5) | | 19(38.0) | 23(34.8) | 25(39.1) | |
| Less than 1 day/month | 6(25.0) | 7(17.1) | 12(11.3) | 1(11.1) | | 19(12.8) | 7(22.6) | | 6(12.0) | 11(16.7) | 9(14.1) | |
| Less than 1 day/week | 2(8.3) | 8(19.5) | 20(18.9) | 0(0.0) | 0.25 | 24(16.1) | 6(19.4) | 0.54 | 4(8.0) | 17(25.8) | 9(14.1) | 0.16 |
| 1-3 days/week | 5(20.8) | 10(24.4) | 34(32.1) | 1(11.1) | | 44(29.5) | 6(19.4) | | 20(40.0) | 13(19.7) | 17(26.6) | |
| 4-7 days/week | 0(0.0) | 3(7.3) | 3(2.8) | 1(11.1) | | 6(4.0) | 1(3.2) | | 1(2.0) | 2(3.0) | 4(6.2) | |

[a]1 missing.

[*]Significant at p<0.05, [**]significant at p<0.01, [***]significant at p<0.001, using ANOVA or student's t-test for normally distributed variables, Kruskal-Wallis or Wilcoxon rank-sum test for nonparametric variables, and Chi-square or Fisher's exact test for categorical variables.

*Abbreviations*: HDP, Hypertensive Disorders of Pregnancy; CH, chronic hypertension; GH, gestational hypertension; PE, pre-eclampsia; CH+PE, pre-eclampsia superimposed on chronic hypertension; BMI, body mass index; SD, standard deviation; IQR, interquartile range.

**Table S4**. Subgroup analysis of women's lifestyle outcomes at 12 months post-HDP stratified by hypertensive pregnancy subtype, body mass index, and study group

| Lifestyle outcomes | HDP subtypes | | | | | Baseline BMI | | | BP$^2$ groups | | | |
|---|---|---|---|---|---|---|---|---|---|---|---|---|
| | CH (n=24) | GH (n=41) | PE (n=106) | PE+CH (n=9) | *p*-Value | <30 kg/m$^2$ (n=149) | ⩾30 kg/m$^2$ (n=31) | *p*-Value | Group 1 (n=50) | Group 2 (n=66) | Group 3 (n=64) | *p*-Value |
| ***Mean ±SD*** | | | | | | | | | | | | |
| Vegetable, serves/day | 2.6±1.4 | 2.7±1.4 | 2.8±1.4 | 3.2±2.0 | 0.67 | 2.8±1.5 | 2.6±1.1 | 0.43 | 2.7±1.4 | 2.6±1.3 | 3.0±1.5 | 0.26 |
| Fruit, serves/day | 1.7±1.2 | 1.7±1.0 | 1.8±0.9 | 1.5±1.0 | 0.85 | 1.9±1.0 | 1.1±0.9 | <0.001*** | 1.8±1.1 | 1.6±0.8 | 1.8±1.1 | 0.58 |
| Motivators to engage in healthy behaviours [a] | 4.1±0.5 | 4.3±0.5 | 4.0±0.6 | 19.9±2.9 | 0.18 | 4.1±0.6 | 4.0±0.7 | 0.35 | 4.1±0.6 | 4.0±0.6 | 4.1±0.6 | 0.70 |
| Barriers to healthy living [a] | 2.5±0.8 | 2.7±0.7 | 2.5±0.6 | 23.6±8.6 | 0.47 | 2.5±0.7 | 2.7±0.7 | 0.10 | 2.5±0.7 | 2.5±0.6 | 2.5±0.7 | 0.87 |
| Satisfaction with food & appearance [b] | 2.7±0.7 | 3.0±0.7 | 3.1±0.8 | 12.4±3.6 | 0.15 | 3.1±0.8 | 2.4±0.5 | <0.001*** | 3.1±0.7 | 3.0±0.8 | 2.9±0.8 | 0.52 |
| ***Median [IQR]*** | | | | | | | | | | | | |
| **Diet** | | | | | | | | | | | | |
| Processed meat, serves/day | 0.14[0.24] | 0.10[0.29] | 0.14[0.29] | 0.07[0.29] | 0.78 | 0.14[0.29] | 0.14[0.22] | 0.07 | 0.14[0.29] | 0.14[0.29] | 0.14[0.29] | 0.97 |
| Fried potatoes, serves/day | 0.14[0.11] | 0.07[0.11] | 0.07[0.11] | 0.07[0.14] | 0.11 | 0.07[0.11] | 0.07[0.14] | 0.93 | 0.07[0.14] | 0.07[0.11] | 0.08[0.11] | 0.34 |
| Salty snacks, serves/day | 0.08[0.14] | 0.00[0.14] | 0.03[0.14] | 0.14[0.29] | 0.26 | 0.07[0.14] | 0.00[0.14] | 0.31 | 0.07[0.14] | 0.02[0.09] | 0.03[0.14] | 0.25 |
| Takeaway food, times/week | 0.73[0.83] | 0.46[1.00] | 0.46[0.94] | 0.46[0.77] | 0.71 | 0.46[0.77] | 0.23[1.00] | 0.10 | 0.46[1.00] | 0.46[1.00] | 0.46[0.77] | 0.75 |
| Soft drinks/Cordials, cups/day | 0.14[1.00] | 0.00[0.14] | 0.00[0.21] | 0.00[0.00] | 0.10 | 0.00[0.14] | 0.00[1.00] | 0.14 | 0.00[0.00] | 0.00[0.14] | 0.00[0.32] | 0.08 |
| Fruit drinks, cups/day | 0.00[0.14] | 0.00[0.07] | 0.00[0.00] | 0.00[0.00] | 0.82 | 0.00[0.00] | 0.00[0.14] | 0.32 | 0.00[0.14] | 0.00[0.14] | 0.00[0.00] | 0.38 |
| Water, cups/day | 7.50[6.00] | 6.00[4.00] | 6.00[4.00] | 8.00[3.00] | 0.35 | 6.00[3.50] | 7.00[4.00] | 0.91 | 6.00[3.00] | 6.00[4.00] | 7.00[4.00] | 0.65 |
| **Physical activity** | | | | | | | | | | | | |
| MVPA [a] | 435[387] | 300[245] | 303[341] | 300[270] | 0.60 | 320[335] | 290[278] | 0.47 | 300[324] | 275[246] | 433[383] | 0.03 |
| Walking [b] | 195[278] | 180[150] | 180[254] | 240[160] | 0.90 | 210[210] | 120[198] | 0.12 | 210[180] | 150[198] | 210[360] | 0.29 |
| Vigorous gardening work | 0[0] | 0[0] | 0[0] | 0[0] | 0.18 | 0[0] | 0[0] | 0.38 | 0[0] | 0[0] | 0[0] | 0.89 |
| Leisure time exercise | 85[210] | 30[180] | 40[128] | 60[90] | 0.69 | 40[150] | 60[163] | 0.74 | 60[174] | 30[139] | 43[180] | 0.55 |

| Lifestyle outcomes | HDP subtypes | | | | | Baseline BMI | | | BP[2] groups | | | |
|---|---|---|---|---|---|---|---|---|---|---|---|---|
| | CH (n=24) | GH (n=41) | PE (n=106) | PE+CH (n=9) | *p*-Value | <30 kg/m$^2$ (n=149) | ⩾30 kg/m$^2$ (n=31) | *p*-Value | Group 1 (n=50) | Group 2 (n=66) | Group 3 (n=64) | *p*-Value |
| ***Frequency (%)*** | | | | | | | | | | | | |
| ⩾5 vegetable serves/day | 3(12.5) | 5(12.2) | 8(7.5) | 2(22.2) | 0.32 | 16(10.7) | 2(6.5) | 0.74 | 5(10.0) | 4(6.1) | 9(14.1) | 0.32 |
| ⩾2 fruit serves/day | 10(41.7) | 22(53.7) | 62(58.5) | 3(33.3) | 0.28 | 91(61.1) | 6(19.4) | <0.001*** | 29(58.0) | 36(54.5) | 32(50.0) | 0.69 |
| Meet both for vegetable and fruit | 1(4.2) | 4(9.8) | 7(6.6) | 1(11.1) | 0.66 | 12(8.1) | 1(3.2) | 0.70 | 5(10.0) | 2(3.0) | 6(9.4) | 0.25 |
| ⩾150 MVPA minutes/week | 21(87.5) | 34(82.9) | 88(83.0) | 8(88.9) | 0.97 | 127(85.2) | 24(77.4) | 0.42 | 43(86.0) | 54(81.8) | 54(84.4) | 0.83 |
| Meet both for nutrition and MVPA | 1(4.2) | 4(9.8) | 7(6.6) | 1(11.1) | 0.66 | 12(8.1) | 1(3.2) | 0.70 | 5(10.0) | 2(3.0) | 6(9.4) | 0.26 |
| Smoking or using e-cigarettes | | | | | | | | | | | | |
| Never | 11(45.8) | 20(50.0) | 68(64.2) | 6(66.7) | | 91(61.5) | 14(45.2) | | 27(55.1) | 47(71.2) | 31(48.4) | |
| Tried a few times | 5(20.8) | 6(15.0) | 21(19.8) | 3(33.3) | 0.08 | 31(20.9) | 4(12.9) | 0.02* | 8(16.3) | 11(16.7) | 16(25.0) | 0.06 |
| Current/previous | 8(33.3) | 14(35.0) | 17(16.0) | 0(0.0) | | 26(17.6) | 13(41.9) | | 14(28.6) | 8(12.1) | 17(26.6) | |
| Alcohol drinking frequency [c] | | | | | | | | | | | | |
| Never | 9(37.5) | 14(34.1) | 31(29.2) | 7(77.8) | | 52(34.9) | 9(29.0) | | 13(26.0) | 24(36.4) | 24(37.5) | |
| Less than 1 day/month | 4(16.7) | 4(9.8) | 10(9.4) | 0(0.0) | | 13(8.7) | 5(16.1) | | 6(12.0) | 6(9.1) | 6(9.4) | |
| Less than 1 day/week | 5(20.8) | 7(17.1) | 21(19.8) | 0(0.0) | 0.39 | 26(17.4) | 7(22.6) | 0.42 | 7(14.0) | 14(21.2) | 12(18.8) | 0.78 |
| 1-3 days/week | 5(20.8) | 13(31.7) | 41(38.7) | 2(22.2) | | 53(35.6) | 8(25.8) | | 22(44.0) | 19(28.8) | 20(31.2) | |
| 4-7 days/week | 1(4.2) | 3(7.3) | 3(2.8) | 0(0.0) | | 5(3.4) | 2(6.5) | | 2(4.0) | 3(4.5) | 2(3.1) | |

[a]22 missing; [b]2 missing; [c]1 missing.

*Significant at p<0.05, **significant at p<0.01, ***significant at p<0.001, using ANOVA or student's t-test for normally distributed variables, Kruskal-Wallis or Wilcoxon rank-sum test for nonparametric variables, and Chi-square or Fisher's exact test for categorical variables.

*Abbreviations*: n, number; HDP, Hypertensive Disorders of Pregnancy; CH, chronic hypertension; GH, gestational hypertension; PE, pre-eclampsia; CH+PE, pre-eclampsia superimposed on chronic hypertension; BMI, body mass index; SD, standard deviation; IQR, interquartile range.

**Table S5.** Subgroup analysis of women's lifestyle outcomes at 3-4 years post-HDP stratified by hypertensive pregnancy subtype, body mass index, and study group

| Lifestyle outcomes | HDP subtypes | | | | | Baseline BMI | | | BP$^2$ groups | | | |
|---|---|---|---|---|---|---|---|---|---|---|---|---|
| | CH (n=24) | GH (n=41) | PE (n=106) | PE+CH (n=9) | *p*-Value | <30 kg/m$^2$ (n=149) | ⩾30 kg/m$^2$ (n=31) | *p*-Value | Group 1 (n=50) | Group 2 (n=66) | Group 3 (n=64) | *p*-Value |
| ***Mean ±SD*** | | | | | | | | | | | | |
| Vegetable, serves/day | 4.1±1.9 | 4.8±2.1 | 4.1±1.9 | 3.7±1.7 | 0.25 | 4.3±2.0 | 4.2±1.8 | 0.81 | 4.2±2.0 | 4.2±2.0 | 4.3±1.9 | 0.91 |
| Fruit, serves/day | 2.0±1.5 | 2.0±1.0 | 2.3±1.3 | 2.0±0.7 | 0.64 | 2.3±1.3 | 1.5±0.9 | <0.001*** | 2.2±1.2 | 2.0±1.3 | 2.3±1.3 | 0.30 |
| Motivators to engage in healthy behaviours [a] | 4.1±0.6 | 4.3±0.6 | 4.1±0.6 | 3.8±0.6 | 0.35 | 4.1±0.6 | 4.1±0.6 | 0.63 | 4.1±0.6 | 4.1±0.6 | 4.2±0.6 | 0.89 |
| Barriers to healthy living [b] | 2.6±0.9 | 2.6±0.7 | 2.4±0.7 | 3.0±0.9 | 0.05 | 2.4±0.7 | 2.9±0.8 | 0.006** | 2.5±0.8 | 2.5±0.6 | 2.5±0.8 | 0.94 |
| Satisfaction with food & appearance [c] | 2.5±0.7 | 2.6±0.7 | 2.8±0.8 | 2.8±0.7 | 0.18 | 2.8±0.8 | 2.3±0.6 | <0.001*** | 2.7±0.7 | 2.8±0.7 | 2.7±0.8 | 0.85 |
| ***Median [IQR]*** | | | | | | | | | | | | |
| **Diet** | | | | | | | | | | | | |
| Processed meat, serves/day | 0.15[0.25] | 0.16[0.19] | 0.13[0.19] | 0.07[0.13] | 0.17 | 0.13[0.19] | 0.16[0.22] | 0.03* | 0.16[0.19] | 0.11[0.19] | 0.15[0.23] | 0.28 |
| Fried potatoes, serves/day | 0.12[0.20] | 0.10[0.13] | 0.15[0.13] | 0.15[0.15] | 0.42 | 0.13[0.13] | 0.18[0.33] | 0.03* | 0.10[0.12] | 0.10[0.11] | 0.18[0.20] | 0.006** |
| Salty snacks, serves/day | 0.53[0.52] | 0.34[0.44] | 0.42[0.41] | 0.37[0.64] | 0.71 | 0.40[0.42] | 0.51[0.63] | 0.19 | 0.38[0.39] | 0.29[0.43] | 0.47[0.47] | 0.10 |
| Takeaway food, times/week | 1.50[0.25] | 0.50[1.00] | 1.50[1.00] | 0.50[1.00] | 0.16 | 1.50[1.00] | 1.50[1.00] | 0.89 | 1.50[1.00] | 0.50[1.00] | 1.50[1.00] | 0.01* |
| Soft drinks/Cordials, cups/day | 0.17[0.96] | 0.08[0.16] | 0.09[0.55] | 0.03[0.06] | 0.09 | 0.08[0.28] | 0.57[0.59] | <0.001*** | 0.09[0.55] | 0.08[0.30] | 0.09[0.57] | 0.65 |
| Fruit drinks, cups/day | 0.02[0.13] | 0.02[0.07] | 0.02[0.14] | 0.07[0.12] | 0.48 | 0.02[0.07] | 0.02[0.10] | 0.74 | 0.02[0.07] | 0.02[0.14] | 0.02[0.07] | 0.74 |
| Water, cups/day | 5.00[5.00] | 5.00[2.00] | 5.00[5.00] | 5.00[2.00] | 0.72 | 5.00[5.00] | 5.00[4.00] | 0.40 | 5.00[1.50] | 5.00[4.25] | 5.00[5.00] | 0.83 |
| **Physical activity** | | | | | | | | | | | | |
| MVPA [d] | 420[730] | 483[644] | 620[710] | 480[390] | 0.49 | 550[690] | 480[670] | 0.59 | 735[694] | 465[610] | 535[740] | 0.40 |
| Walking [e] | 190[308] | 210[330] | 210[250] | 180[150] | 0.82 | 210[260] | 165[353] | 0.48 | 240[365] | 180[210] | 200[280] | 0.44 |
| Vigorous gardening work [f] | 0[0] | 0[70] | 0[0] | 0[30] | 0.07 | 0[0] | 0[25] | 0.70 | 0[0] | 0[28] | 0[10] | 0.87 |
| Leisure time exercise [f] | 40[124] | 0[120] | 30[120] | 0[40] | 0.65 | 0[120] | 0[158] | 0.78 | 35[173] | 0[120] | 0[105] | 0.67 |

| Lifestyle outcomes | HDP subtypes | | | | | Baseline BMI | | | BP² groups | | | |
|---|---|---|---|---|---|---|---|---|---|---|---|---|
| | CH (n=24) | GH (n=41) | PE (n=106) | PE+CH (n=9) | *p*-Value | <30 kg/m² (n=149) | ⩾30 kg/m² (n=31) | *p*-Value | Group 1 (n=50) | Group 2 (n=66) | Group 3 (n=64) | *p*-Value |
| ***Frequency (%)*** | | | | | | | | | | | | |
| ⩾5 vegetable serves/day | 8(33.3) | 17(41.5) | 36(34.0) | 3(33.3) | 0.84 | 53(35.6) | 11(35.5) | 1.00 | 16(32.0) | 23(34.8) | 25(39.1) | 0.73 |
| ⩾2 fruit serves/day | 9(37.5) | 22(53.7) | 58(54.7) | 4(44.4) | 0.50 | 85(57.0) | 8(25.8) | 0.003** | 25(50.0) | 32(48.5) | 36(56.2) | 0.65 |
| Meet both for vegetable and fruit | 6(25.0) | 11(26.8) | 28(26.4) | 2(22.2) | 1.00 | 40(26.8) | 7(22.6) | 0.79 | 10(20.0) | 18(27.3) | 19(29.7) | 0.49 |
| ⩾150 MVPA minutes/week | 15(71.4) | 31(91.2) | 89(88.1) | 8(88.9) | 0.18 | 120(88.2) | 23(79.3) | 0.33 | 39(88.6) | 52(85.2) | 52(86.7) | 0.88 |
| Meet both for nutrition and MVPA | 4(19.0) | 9(26.5) | 27(26.7) | 2(22.2) | 0.95 | 36(26.5) | 6(20.7) | 0.68 | 9(20.5) | 16(26.2) | 17(28.3) | 0.65 |
| Smoking or using e-cigarettes | | | | | | | | | | | | |
| Never | 14(58.3) | 19(46.3) | 66(62.9) | 8(88.9) | | 90(60.8) | 17(54.8) | | 30(61.2) | 42(63.6) | 35(54.7) | |
| Tried a few times | 4(16.7) | 9(22.0) | 22(21.0) | 1(11.1) | 0.19 | 33(22.3) | 3(9.7) | 0.05 | 10(20.4) | 16(24.2) | 10(15.6) | 0.15 |
| Current/previous | 6(25.0) | 13(31.7) | 17(16.2) | 0(0.0) | | 25(16.9) | 11(35.5) | | 9(18.4) | 8(12.1) | 19(29.7) | |
| Alcohol drinking frequency [c] | | | | | | | | | | | | |
| Never | 11(45.8) | 7(17.1) | 24(22.9) | 6(66.7) | | 40(27.0) | 8(25.8) | | 12(24.5) | 18(27.3) | 18(28.1) | |
| Less than 1 day/month | 3(12.5) | 13(31.7) | 25(23.8) | 1(11.1) | | 34(23.0) | 8(25.8) | | 9(18.4) | 17(25.8) | 16(25.0) | |
| Less than 1 day/week | 7(29.2) | 8(19.5) | 18(17.1) | 0(0.0) | 0.01* | 23(15.5) | 10(32.3) | 0.12 | 8(16.3) | 13(19.7) | 12(18.8) | 0.92 |
| 1-3 days/week | 1(4.2) | 9(22.0) | 32(30.5) | 1(11.1) | | 40(27.0) | 3(9.7) | | 16(32.7) | 14(21.2) | 13(20.3) | |
| 4-7 days/week | 2(8.3) | 4(9.8) | 6(5.7) | 1(11.1) | | 11(7.4) | 2(6.5) | | 4(8.2) | 4(6.1) | 5(7.8) | |

[a]37 missing; [b]3 missing; [c]2 missing; [d]15 missing; [e]6 missing; [f]4 missing.

*Significant at p<0.05, **significant at p<0.01, ***significant at p<0.001, using ANOVA or student's t-test for normally distributed variables, Kruskal-Wallis or Wilcoxon rank-sum test for nonparametric variables, and Chi-square or Fisher's exact test for categorical variables.

*Abbreviations*: n, number; HDP, Hypertensive Disorders of Pregnancy; CH, chronic hypertension; GH, gestational hypertension; PE, pre-eclampsia; CH+PE, pre-eclampsia superimposed on chronic hypertension; BMI, body mass index; SD, standard deviation; IQR, interquartile range.

**Table S6.** Sensitivity analysis of physical activity status between study participants included and excluded at 3-4 years postpartum due to no diet reports from the Australia Eating Survey.

| **Physical activity,** median [IQR] | **Total** (n=255) | ***p*-Value** Overall timepoint effect | **Participants** (n=180) | **Excluded** (n=75) | ***p*-Value** Between-group analysis | ***p*-Value** Group interaction effect |
|---|---|---|---|---|---|---|
| **MVPA [a], minutes/week [a]** | | | | | | |
| 6-months | 300[300] | | 330[280] | 240[380] | 0.03 | |
| 12-months | 300[318] | <0.001*** | 312[331] | 240[272] | 0.007 | <0.001*** |
| 3-4 years | 615[705] | | 515[690] | 715[878] | 0.04 | |
| Walking [b], minutes/week [b] | | | | | | |
| 6-months | 180[205] | | 210[176] | 105[172] | <0.001*** | |
| 12-months | 170[220] | 0.002** | 180[226] | 120[180] | 0.02 | 0.04* |
| 3-4 years | 210[275] | | 198[268] | 225[296] | 0.52 | |
| Vigorous gardening work [c] | | | | | | |
| 6-months | 0[0] | | 0[0] | 0[0] | 0.23 | |
| 12-months | 0[0] | <0.001*** | 0[0] | 0[0] | 0.49 | 0.26 |
| 3-4 years | 0[30] | | 0[20] | 0[80] | 0.05 | |
| Leisure time exercise [c] | | | | | | |
| 6-months | 15[120] | | 30[135] | 0[90] | 0.08 | |
| 12-months | 25[120] | 0.78 | 40[150] | 0[74] | 0.003 | 0.28 |
| 3-4 years | 0[120] | | 0[120] | 0[90] | 0.23 | |

[a]27 missing; [b]13 missing; [c]7 missing.

*Significant at p<0.05, **significant at p<0.01, ***significant at p<0.001.

*Abbreviations*: IQR, interquartile range.
